# Insights Gained From an Ultrarare Case of Progressive Osseous Heteroplasia With Severe Complications

**DOI:** 10.7759/cureus.78191

**Published:** 2025-01-29

**Authors:** Ana Coimbra, Adriana Henriques, Joana Duarte, Jandira Lima, Sara Leitão

**Affiliations:** 1 Internal Medicine, Coimbra Local Health Unit (ULS Coimbra), Coimbra, PRT

**Keywords:** clinical decision-making, gnas mutations, heterotopic ossification, intramembranous ossification, personalized medicine, progressive osseous heteroplasia, rare bone disorders, ultrarare disorders

## Abstract

Progressive osseous heteroplasia (POH) is an exceptionally rare genetic disorder characterized by progressive heterotopic ossification of cutaneous, subcutaneous, and deep connective tissues. Most cases are associated with paternally inherited inactivating mutations in the guanine nucleotide-binding protein, alpha-stimulating gene. However, the genetic basis remains undefined in a subset of patients, adding further complexity to its pathogenesis. With fewer than 100 documented cases worldwide, POH presents significant diagnostic and therapeutic challenges, compounded by its rarity and clinical heterogeneity. Typically manifesting in infancy with dermal ossification, the disorder progresses to involve deeper structures, leading to ankylosis, deformities, and profound functional limitations. Unlike other ossification disorders, such as fibrodysplasia ossificans progressiva and Albright’s hereditary osteodystrophy, POH is characterized by intramembranous ossification and a distinct lack of skeletal malformations.

We report the case of an adult male with unilateral POH, a particularly unusual presentation, further complicated by septic arthritis, progressing to shock and multiorgan dysfunction. The patient initially presented with severe hip pain and functional impairment. Imaging confirmed septic arthritis, and conservative management with medical therapy and long-term antibiotics was initiated. Despite these measures, his condition deteriorated, culminating in septic shock and organ failure. The absence of established guidelines for POH necessitated careful interdisciplinary deliberation. Surgical intervention, including debridement, proximal femoral osteotomy, and placement of an antibiotic-impregnated spacer, was performed. A second-stage total hip arthroplasty restored mobility and resolved systemic complications, resulting in significant functional recovery.

This case underscores the significant challenges posed by ultra-rare diseases like POH, where clinical management is often hindered by the absence of evidence-based protocols or precedent. The reliance on individualized decision-making highlights the importance of careful documentation and reporting of such cases to guide future clinical practice. Furthermore, the complexity and unpredictability of POH underscore the need for collaborative research efforts aimed at developing robust diagnostic criteria, therapeutic strategies, and long-term management guidelines for this debilitating condition.

## Introduction

Progressive osseous heteroplasia (POH) is an ultrarare genetic autosomal disorder characterized by progressive heterotopic ossification. Initially manifesting in the dermis and subsequently advancing into deeper connective tissues, POH typically presents during infancy and poses significant diagnostic and management challenges due to its rarity and severity [1-7].

First described in 1994 by Kaplan et al. [8], POH is thought to be caused by heterozygous inactivating mutations of the guanine nucleotide-binding protein, alpha-stimulating (GNAS) gene, which encodes the alpha subunit of the G-stimulatory protein of adenylyl cyclase (Gsα) [2,4,9]. This condition falls within the broader category of inactivating parathyroid hormone (PTH)/parathyroid hormone-related protein signaling disorders [9,10] unified by mutations affecting the Gsα protein, which plays a crucial role in mesenchymal differentiation and is essential for the proper signaling in bone development and regulation. POH overlaps symptomatically with other GNAS mutation disorders, such as Albright's hereditary osteodystrophy (AHO) and pseudohypoparathyroidism. Yet, it follows a distinct clinical trajectory marked by severe and pervasive ossification, normal endocrine function, and no congenital malformations. POH generally exhibits significant early-onset and more extensive ossification, underscoring it as a more severe form of GNAS-related disorders [2,4,7,9].

Diagnosis is based on clinical criteria, including the presence of ectopic bone formation without the full spectrum of AHO features, the absence of hormone resistance, such as to PTH, and the absence of skeletal malformations [3,4,7]. Radiographic evaluations often reveal a distinct reticular pattern of ossification that correlates with the clinical observation of palpable masses. Histopathological examination supports the diagnostic process by revealing irregular woven bone spicules, confirming intramembranous ossification, a hallmark of POH [2-4,7].

The complexity of POH extends beyond its clinical presentation to its genetic underpinnings. Once considered a Mendelian autosomal dominant trait linked to GNAS gene mutations, the diversity in clinical manifestations and the rarity of familial occurrences have prompted a revision of the genetic model [1,9,11]. Recent insights suggest a nuanced mechanism of superimposed mosaicism. This hypothesis proposes that POH may result from a postzygotic loss of heterozygosity, leading to segmental manifestations that complicate the genetic landscape and significantly impact genetic counseling and disease understanding [4,6,12].

Current management of POH focuses on symptomatic relief, as there is no cure. The condition can vary in progression and severity, and treatments may include physical therapy and surgical interventions for severe cases [1,3,5,7]. Managing POH poses significant challenges due to the relentless progression of ossification. Surgical interventions often result in recurrence [2,3,13], and no effective treatment currently exists, underscoring a critical need for genetic research to unravel the molecular mechanisms underpinning POH to devise effective treatments. Research into the molecular pathways affected by GNAS mutations suggests potential therapeutic targets [7,8]. For example, hedgehog (Hh) signaling inhibitors, currently used in cancer therapies, show promise for treating conditions like POH due to their role in modulating abnormal bone formation [14]. Cong et al. [15] recently described a self-amplifying, self-propagating loop of yes-associated protein (YAP) and Sonic hedgehog (SHH) that plays a key molecular role in the development of ossification.

The current report aims to enhance the clinical discourse on POH by detailing a case characterized by severe, life-threatening complications where surgical intervention proved pivotal. It contributes valuable insights into the strategic management of complex manifestations associated with POH, emphasizing the importance of collaborative team approaches and multidisciplinary discussions coupled with rigorous clinical reasoning in contexts devoid of established guidelines or substantial experiential knowledge. These findings suggest that in cases of severe complications necessitating urgent surgical management, timely intervention may be critical and should not be unnecessarily delayed in patients with this condition. However, further investigation is warranted to substantiate this approach.

This paper explores the clinical presentation and management challenges of this unique case of POH. It offers a detailed examination of the decision-making process related to surgical interventions and their role in managing severe heterotopic ossification. The insights from this case are poised to enhance the understanding of POH's clinical spectrum and inform future therapeutic strategies, aiming to guide clinicians toward more effective management of this perplexing condition.

## Case presentation

**Figure 1:**
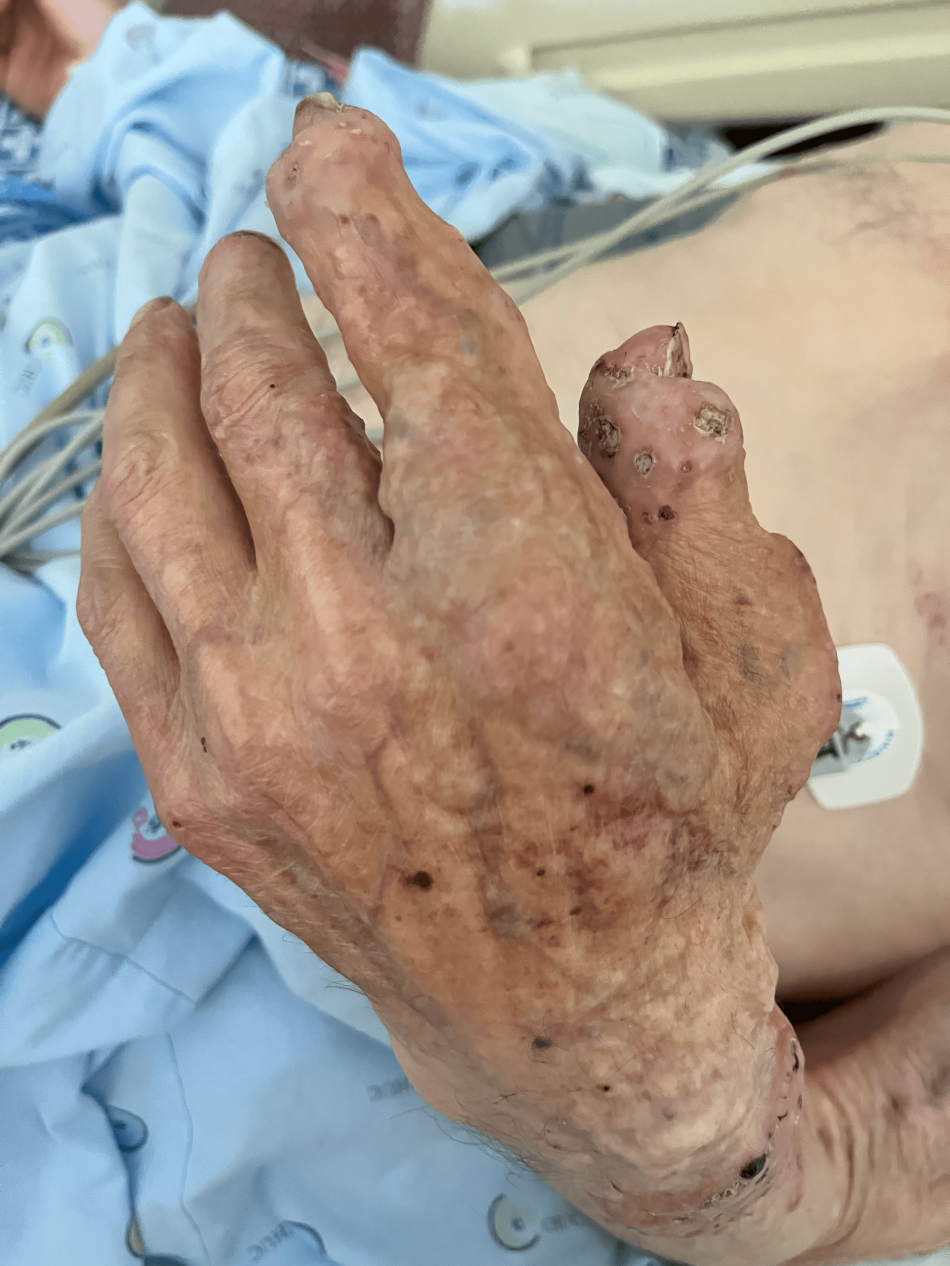
Unilateral presentation of POH Subcutaneous nodules and plaques present as firm, gritty-textured lesions unilaterally affecting the left side of the patient's trunk and limbs. These lesions, first noted in early childhood, exhibited progressive ossification and are characteristic of POH POH: progressive osseous heteroplasia

A 68-year-old male patient with a history of small-vessel vasculitis, arterial hypertension, atrial fibrillation, dyslipidemia, and hyperthyroidism secondary to amiodarone use presented to the emergency department with complaints of palpitations, pain, and functional impairment in his right coxofemoral joint. He reported that these symptoms began following a fall two weeks before. His clinical background also included a longstanding history of unilateral cutaneous osteomas on the left side (Figure 1), first noted in childhood and of unspecified cause, an alcohol abuse disorder now in remission, and a femur fracture at age 45. He was currently on a regimen of antihypertensives, beta-blockers, statins, and anticoagulants, and lived in a rural area with his wife and four children.

Upon admission, the patient presented with tachycardia and epistaxis. Examination revealed macroscopic hematuria, petechiae on his limbs, and widespread firm subcutaneous nodules and plaques with a gritty texture, affecting only the left side of the trunk and limbs. A significant inflammatory response was indicated by an elevated C-reactive protein level of 39.48 mg/dL, prompting his admission for further evaluation.

**Figure 2:**
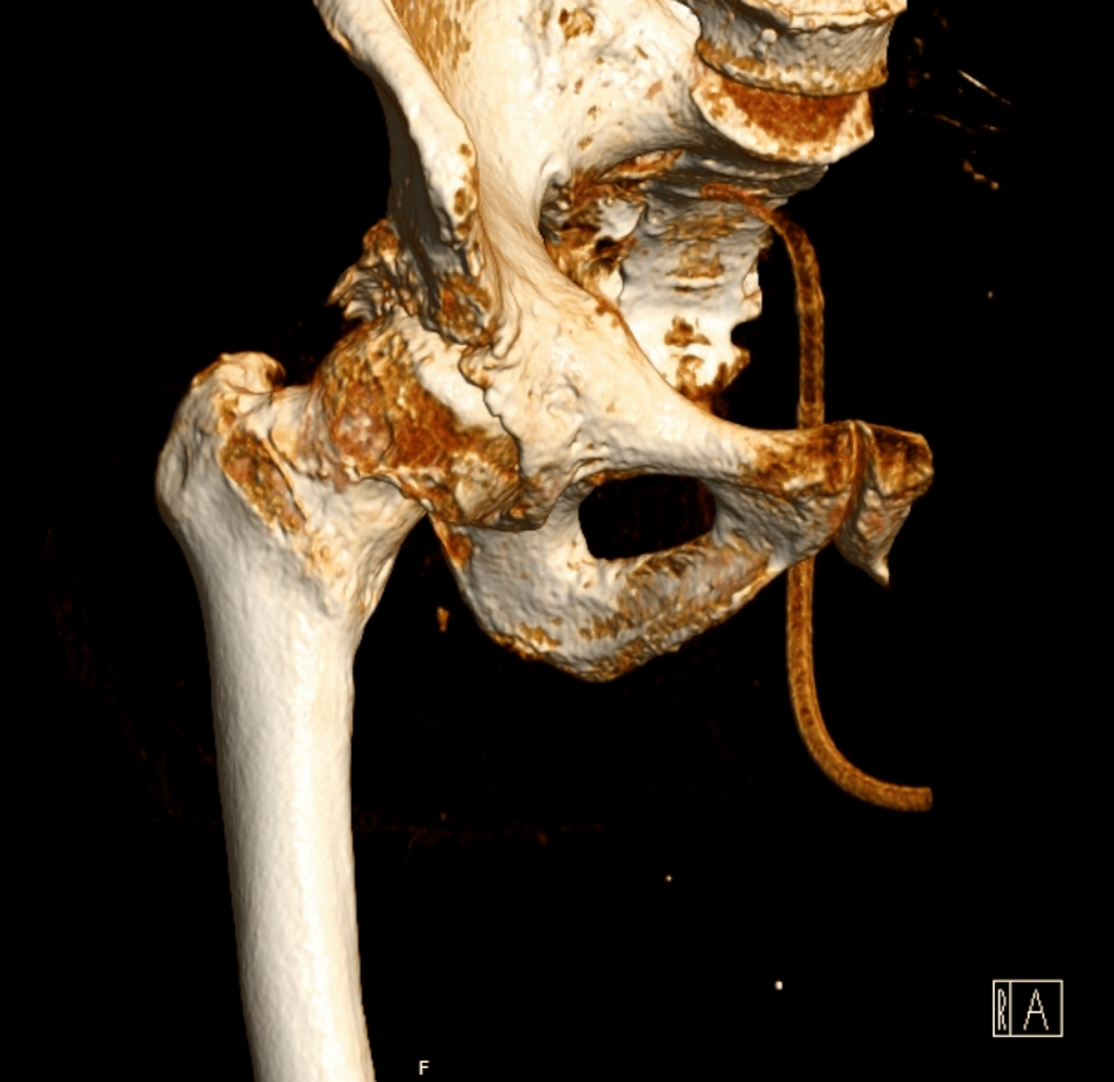
Radiographic features of hip joint in POH CT scan of the right hip showed advanced coxarthrosis with reduced joint space, subchondral cysts, and osteophytosis, accompanied by joint effusion. These findings are consistent with the severe joint involvement observed in POH and reflect the impact of heterotopic ossification on joint integrity POH: progressive osseous heteroplasia

A CT scan of the right hip was performed (Figure 2) to assess his joint symptoms, revealing exuberant coxarthrosis characterized by reduced joint space, subchondral cysts, osteophytosis, and joint effusion, with no signs of fractures.

Laboratory tests showed normal serum and urine levels of calcium and phosphate, as well as normal levels of parathyroid and thyroid hormones and vitamin D metabolites. Lactate dehydrogenase and alkaline phosphatase levels were also within normal ranges.

A skin biopsy was performed to investigate the nature of the skin lesions, revealing well-differentiated bone formations in the reticular dermis, consistent with osteoma cutis. Radiological examinations of the skeletal system, particularly in the affected areas, showed extensive ossification extending into subcutaneous and deep tissues, particularly severe around the ankle and continuous within the muscular plane. Genetic testing on peripheral blood did not identify mutations in the GNAS gene.

The final diagnosis was unilateral POH, based on clinical manifestations, symptom progression, normal phosphocalcium metabolism, skin biopsy results, and radiological findings.

During hospitalization, the patient was treated for a urinary tract infection that exacerbated his atrial fibrillation, initially with ceftriaxone and clindamycin, followed by trimethoprim-sulfamethoxazole due to insufficient initial response. His hospital course was further complicated by hypoxemic respiratory insufficiency, requiring temporary oxygen supplementation, and resolved infections of the oral mucosa and left hallux. Pain and functional impairment of the right lower limb were progressively managed with analgesia, enabling him to begin ambulation and gait training, which he tolerated well. He was discharged, capable of walking with the aid of a walker, and directed to a continued care unit for ongoing management.

After discharge to the care unit, the patient's condition markedly deteriorated; he experienced escalating pain, altered mental status, and dizziness, which substantially hindered his rehabilitation efforts. Over subsequent weeks, he became bedridden and entirely dependent, developed pressure ulcers, and suffered from reactive depression. Severe anemia was identified during routine laboratory work at the care unit, with a hemoglobin level of 7.5 g/dL, prompting his referral to the local emergency department for potential transfusional support.

Subsequent imaging at this facility revealed pronounced synovitis of the right hip. Given the clinical presentation, septic arthritis was suspected. The CT scan showed significant intra-articular effusion, a widening of the articular line by 12 mm at the lower and medial levels, sclerosis of the articular surfaces, and erosive foci. Notably, there was hypoechoic infiltration of the periarticular soft tissues, suggesting an inflammatory or infectious process, most severe at the anteroinferior and peritrochanteric levels.

Orthopedic consultants recommended conservative management with long-term suppressive antibiotic therapy, considering the patient's complex history of POH and his bedridden state, which compounded the risks associated with surgical interventions. He was discharged back to the continued care unit. Despite these measures, the patient's condition continued to deteriorate, eventually presenting with signs of a systemic inflammatory response, including hypotension and persistent tachycardia. This clinical decline warranted an urgent referral to our emergency service, in which a comprehensive clinical review underscored the ongoing risk of sepsis. Given the clinical picture suggesting worsening septic arthritis of the right hip under outpatient antibiotic therapy, further radiographic examination was ordered and demonstrated progression of coxarthrosis with notable articular surface destruction. An orthopedic evaluation was requested, leading to an interdisciplinary consultation and a thorough reassessment in which a decision was made to proceed with surgical intervention to address what was now considered a likely septic focus in the hip.

**Figure 3:**
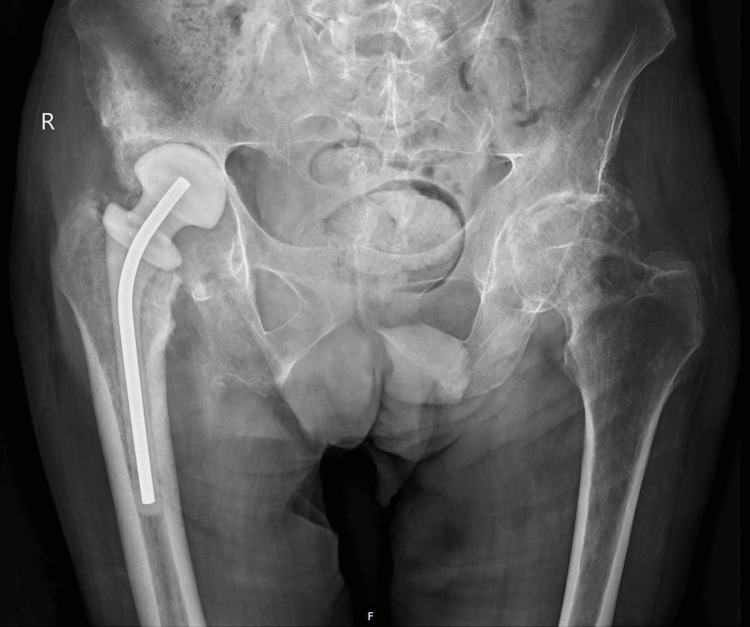
Postoperative radiographic study following proximal femoral osteotomy Radiographic image obtained after proximal femoral osteotomy, demonstrating the placement of antibiotic-impregnated spacers to manage infection and maintain joint spacing. This intervention represents a critical step in addressing septic arthritis associated with POH POH: progressive osseous heteroplasia

The surgical intervention involved meticulous cleaning and disinfection of the hip joint. During the procedure, hemopurulent periarticular fluid was aspirated and sent for microbiological analysis, subsequently identifying methicillin-resistant *Staphylococcus aureus* (MRSA). Additionally, fragments from the femoral head, femoral canal, periarticular tissues, and acetabulum were cultured, all of which confirmed the presence of MRSA. A proximal femoral osteotomy was performed, followed by the placement of gentamicin and vancomycin-impregnated spacers to manage infection and maintain joint spacing (Figure 3). The procedure was completed without any complications, and the patient was returned to the care unit for recovery.

At a four-month follow-up, the patient demonstrated significant improvement; he reported no significant pain and was capable of ambulating with the aid of two walkers. He had gained weight and showed no signs of inflammation, indicating a successful response to the surgical and antibiotic management.

**Figure 4:**
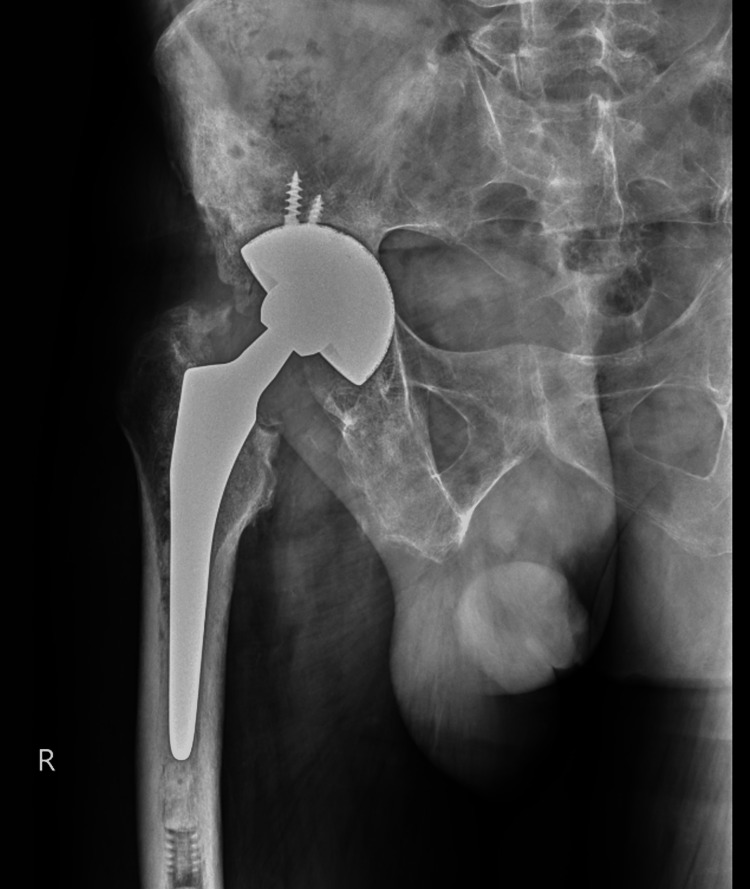
Final radiographic study after right hip arthroplastic reconstruction

Ten months after the initial operation and with continued clinical improvement, a second surgical procedure was performed: right hip arthroplastic reconstruction. The previously placed spacers were removed, followed by surgical washing and debridement. The reconstruction then involved the implantation of a hybrid dual-mobility total hip prosthesis, which included a multihole cup and a cemented femoral stem with a dual-mobility head (Figure 4). During this procedure, samples were once again collected from the femoral head, femoral canal, periarticular tissues, acetabulum, and synovial fluid for microbiological analysis; all cultures returned negative results, confirming the resolution of the infection.

At the three-month follow-up subsequent to the secondary surgical intervention, the patient demonstrated a complete restoration of functional capacity, mirroring his premorbid state. He experienced no residual pain and exhibited substantial mobility, requiring only minimal assistance for ambulation. Furthermore, he had successfully resumed his occupational activities. No postoperative complications were observed, and no additional medical interventions were deemed necessary. The patient’s functional capacity was quantitatively assessed using the Katz index of independence in activities of daily living (Katz ADL) at two critical points: before the first surgical intervention and at the three-month follow-up after the second surgery. Initially, a Katz ADL score of zero was recorded, reflecting total dependence and an inability to perform basic self-care tasks due to debilitating pain, systemic infection, and immobility. By contrast, at the three-month postoperative evaluation, the patient achieved a Katz ADL score of 6, indicative of full recovery of self-care abilities and independent functioning.

A concise timeline summarizing key interventions and outcomes is presented in Table 1, providing an overview of the patient’s clinical course and management over 13 months.

**Table 1:** Timeline of major interventions and clinical outcomes Timeline summarizing key interventions and outcomes for the patient, highlighting major clinical events, diagnostic evaluations, treatments, and follow-up milestones over the course of 13 months POH: progressive osseous heteroplasia; MRSA: methicillin-resistant *S. aureus*

Time	Event	Observations
Admission	Emergency department visit	Complaints of palpitations, right hip pain, and functional impairment. An initial diagnostic workup initiated
Day 5	Inpatient evaluation and diagnosis	POH diagnosis was established based on clinical, radiological, and histopathological findings
Month 1	Discharge to continued care unit	Persistent pain, anemia, and systemic symptoms were noted, with limited rehabilitation progress
Month 2	Reevaluation for worsening symptoms	Synovitis and suspected septic arthritis were identified on CT. Conservative management initiated
Month 3	Referral to emergency department	Systemic inflammatory response observed; surgical intervention deemed necessary after interdisciplinary review
	First surgical intervention	Hip joint debridement, spacer placement, and MRSA identification. Positive cultures from multiple samples
Month 7	Follow-up evaluation	Marked clinical improvement with reduced pain, weight gain, and ambulation using two walkers
Month 10	Second surgical intervention	Spacer removal and right hip arthroplasty; all cultures returned negative
Month 13	Follow-up evaluation	Full functional recovery achieved; no residual pain; resumed occupational activities

## Discussion

This case of POH showcases features that are emblematic and atypical of the disorder. Consistent with documented instances, the patient demonstrated extensive and progressive heterotopic ossification. Uniquely, he developed severe synovitis progressing to septic arthritis, an uncommon complication within the context of POH. This illustrates the unpredictable severity and heterogeneous nature of the disease [3,16]. This case underscores the profound challenges posed by POH, a rare and unpredictable disorder, particularly when compounded by atypical complications that are not well-characterized in current literature.

Existing knowledge on POH focuses primarily on its ossification patterns and physical manifestations, with minimal attention to its potential systemic vulnerabilities, such as an increased risk of infections or inflammatory triggers. Notably, the unilateral manifestation of cutaneous and subcutaneous ossifications from early childhood typifies the classic presentation of POH. However, the development of severe septic arthritis in the contralateral hip, devoid of superficial osteomas, represents an atypical clinical progression. Additionally, a published case report identifies an inflammatory event as a potential trigger for heterotopic bone formation [5]. However, the absence of traumatic or inflammatory triggers has been noted to assist in differentiating POH from other disorders associated with heterotopic bone formation. This deviation underscores a potentially complex interaction between systemic and localized pathophysiological processes in POH, suggesting broader impacts of the disease than previously recognized.

The management of POH in this patient contrasted sharply with standard accounts that primarily describe isolated ossifications with no mention of severe joint infections. While the literature often focuses on managing ossification and its physical limitations [13], this case was pivotal in highlighting infectious complications.

The decision-making process, in this case, transitioning from conservative management to surgical intervention, reflects the intricate considerations necessary in managing complex and rare cases. It emphasizes the importance of vigilant monitoring for severe, potentially overlooked complications like infection, which can significantly alter therapeutic approaches and patient outcomes. It highlights the necessity of a multidisciplinary strategy in managing such intricate cases, integrating expertise across orthopedics, internal medicine, rehabilitation medicine, and infectious diseases.

Importantly, the lessons from this case emphasize that life-saving surgical interventions should not be delayed when severe complications necessitate urgent management. While the rarity and complexity of POH may complicate decision-making, this case suggests that timely surgical action, when warranted, can result in significant clinical improvement and favorable outcomes, even in the context of this challenging disorder.

From a research standpoint, this case hints at an underexplored link between heterotopic ossification and an increased risk of joint infections, suggesting new avenues for understanding POH pathophysiology and potential therapeutic targets. By unraveling the complex pathogenesis of POH and gaining a deeper understanding of the molecular mechanisms driving the transformation of skin, fat, and muscle precursor cells into bone, there is hope for unlocking new insights that could illuminate and ultimately improve the treatment of more common osteogenic disorders [3,17].

One promising avenue lies in the Hh signaling pathway, which plays a critical role in regulating bone formation and is closely tied to the dysregulated osteogenesis observed in POH. Preclinical research has identified a self-amplifying loop involving SHH and YAP activity that drives ectopic bone formation in conditions such as POH. Targeting this pathway with Hh inhibitors, already being explored in oncology and fibrotic disorders, holds potential as a novel therapeutic strategy to mitigate the progression of heterotopic ossification and its associated complications.

Integrating such innovative approaches into clinical practice could complement surgical and symptomatic management, address the molecular underpinnings of the disease, and potentially reduce the risk of severe outcomes. This highlights the importance of translational research in bridging the gap between preclinical findings and real-world applications, particularly for rare disorders like POH. By advancing targeted therapies and fostering multidisciplinary collaboration, this case underscores the potential to improve outcomes for POH and a broader spectrum of osteogenic disorders influenced by similar pathways.

Furthermore, the successful outcome following the initial conservative treatment failure highlights the importance of carefully timed surgical interventions in managing complications associated with POH. This case underscores the necessity of personalized treatment strategies tailored to the unique progression and clinical challenges presented by each patient and advocates for continuous evaluation and adaptation of management plans based on evolving clinical circumstances. While this case offers valuable insights, its generalizability is inherently limited due to the rarity of POH and the absence of broader data. Nevertheless, it emphasizes the critical need for collecting systematic clinical data and developing comprehensive registries for POH patients to inform the creation of evidence-based treatment guidelines.

Future implications of this case extend to developing therapeutic frameworks that integrate molecular advances, such as targeting dysregulated signaling pathways, with personalized surgical and supportive interventions. This approach could serve as a template for managing other ultrarare conditions with similarly complex presentations. Additionally, the ethical considerations inherent in treating ultrarare disorders, particularly the challenges of decision-making in the absence of established guidelines, merit reflection. In cases like this, multidisciplinary collaboration and transparent communication with patients and their families are essential to navigating uncertainties and ensuring that care decisions align with the best available evidence and the patient’s goals and values. Ultimately, this case highlights the potential to refine management strategies through the convergence of individualized care, emerging therapeutic advancements, and ethical deliberation.

## Conclusions

This case of POH demonstrates both typical features, such as unilateral heterotopic ossification from early childhood, and rare complications, including septic arthritis in a contralateral joint devoid of superficial osteomas. The successful surgical intervention not only resolved systemic infection and septic arthritis but also restored functional mobility, with the patient regaining independence as indicated by the Katz ADL. These outcomes highlight the importance of prompt multidisciplinary decision-making and tailored management strategies in addressing severe, life-threatening complications of POH.

While this case underscores the potential benefits of surgical intervention in appropriately selected scenarios, its generalizability is inherently limited by the rarity of POH and the unique circumstances of this patient. Further research is needed to explore systemic vulnerabilities in POH that may predispose patients to such complications and to define evidence-based guidelines for balancing conservative and surgical approaches. This case underscores the critical role of team-based care, combining orthopedic expertise, infectious disease management, and rehabilitation, in navigating the complexities of managing rare and challenging conditions like POH.
